# The molecular pathogenesis of Trichilemmal carcinoma

**DOI:** 10.1186/s12885-020-07009-7

**Published:** 2020-06-03

**Authors:** Jeong Hyun Ha, Cheol Lee, Kyu Sang Lee, Chang-sik Pak, Choong-Hyun Sun, Youngil Koh, Hak Chang

**Affiliations:** 1 Department of Plastic and Reconstructive Surgery, Seoul Metropolitan Government-Seoul National University Boramae Medical Center, Seoul, Korea; 2 Department of Pathology, Seoul National University Hospital, Seoul, Korea; 3 Department of Pathology, Seoul National University Bundang Hospital, Gyeonggi, Korea; 4 Department of Plastic and Reconstructive Surgery, Asan Medical Center, Seoul, Korea; 5 GenomeOpinion, Seoul, Korea; 6 Department of Internal Medicine, Seoul National University Hospital, Seoul, Korea; 7 Cancer Research Institute, Seoul National University College of Medicine, Seoul, Korea; 8 Center for Medical Innovation, Seoul National University Hospital, Seoul, Korea; 9 Department of Plastic and Reconstructive Surgery, Seoul National University College of Medicine, Seoul, Korea

**Keywords:** Trichilemmal carcinoma, DNA sequencing, Molecular pathogenesis

## Abstract

**Background:**

Trichilemmal carcinoma (TC) is an extremely rare hair follicle tumor. We aimed to explore the genetic abnormalities involved in TC to gain insight into its molecular pathogenesis.

**Methods:**

Data from patients diagnosed with TC within a 12-year period were retrospectively reviewed. Genomic DNA isolated from a formalin-fixed paraffin-embedded (FFPE) tumor tissue block was sequenced and explored for a panel of cancer genes.

**Results:**

DNA was extracted from the FFPE tissue of four patients (50% female; mean age, 51.5 years) diagnosed with TC for analysis. The tumor was located in the head and neck of three patients and in the shoulder of one patient. *TP53* mutations (p.Arg213*, p.Arg249Trp, and p.Arg248Gln) were found in three patients. Fusions previously identified in melanoma were detected in two patients (*TACC3-FGFR3* and *ROS1-GOPC* fusions). Other mutations found included NF1-truncating mutation (Arg1362*), *NRAS* mutation (p.Gln61Lys), *TOP1* amplification, and *PTEN* deletion. Overall, genetic changes found in TC resemble that of other skin cancers, suggesting similar pathogenesis. All patients with *TP53* mutations had aggressive clinical course, two who died (OS 93 and 36 months), and one who experienced recurrent relapse.

**Conclusions:**

We reported the genomic variations found in TC, which may give insight into the molecular pathogenesis. Overall, genetic changes found in TC resembled that of other skin cancers, suggesting similar pathogenesis. TP53 mutations was were identified in patients who had an aggressive clinical course. Genetic alterations identified may further suggest the potential treatment options of TC.

## Background

Trichilemmal carcinoma (TC) is a rare hair follicle tumor originating from the outer root sheath epithelium. Although its incidence rate is not clear, it is known to be very rare. According to the review by Hamman et al. [1], only 103 cases has been reported as of 2014. TC was first reported by Headington in 1976 [2] and is also known as tricholemmal carcinoma or tricholemmocarcinoma. It is sometimes confused with the malignant form of a proliferating trichilemmal cyst or proliferating trichilemmal carcinoma, but they have distinct features. TC occurs in aged people as a solitary lesion in a sun-exposed area, especially the head and neck region. Its clinical features resemble basal cell carcinoma, squamous cell carcinoma, keratoacanthoma, or proliferating pilar cyst [3, 4]. It manifests as a polypoid or exophytic mass with or without ulceration. It can be also found as an erythematous or keratotic nodule or indurated plaque. It is thought to be locally aggressive but has a benign clinical course, which can be easily treated with complete excision. Although rare, multiple cases of local recurrence in TC have been reported. In addition, one metastatic tumor has been reported and was treated with adjuvant chemotherapy after wide surgical excision [1]. Typical histopathology of TC shows trichilemmal keratinization and a peripheral palisading pattern indicating the follicular root sheath origin of the tumor. The tumor consists of cytologically atypical, glycogen-rich cells with atypical mitosis. The border is sharply defined and has pushing margins continuous with epidermis or pilosebaceous structures [5].

Its pathogenesis is not well understood, but several factors have been suggested as risk factors including UV and ionizing radiation or preexisting scars. Some reports have described trichilemmal carcinoma diagnosed in patients with xeroderma pigmentosa, those with Cowden syndrome, and solid-organ transplant recipients [6]. Other reports have described TC arising from long-standing seborrheic keratosis [7], wherein histology of the lesion revealed varying degrees of local actinic damage. These reports imply that the development of TC is associated with chronic UV and radiation exposure or aging process. However, other reports have indicated that p53 gene is related with malignant transformation of TC. Takata et al. [8] suggested that the loss of wild-type p53 heterozygosity is a critical event for the malignant transformation of TC. To our knowledge, no other reports have identified the genetic alteration observed in TC.

Genomic profiling is widely performed in various tumors, and the results usually suggest the pathogenesis or treatment options. We aimed to explore the genetic abnormalities involved in TC using panel of cancer genes to obtain insight into its molecular pathogenesis. The results of this study may further suggest possible treatment options or potential drug targets.

## Methods

Clinical data was retrospectively reviewed in six patients who were diagnosed with trichilemmal carcinoma from 2004 to 2016 in Seoul National University Hospital (SNUH) and Seoul National University Bundang Hospital (SNUBH). Of these, four patients underwent a panel sequencing of 83 genes. Other patients were not available for DNA extraction due to the lack of tissue from the formalin-fixed paraffin-embedded (FFPE) tumor tissue block. Overall survival (OS) was calculated as the time from diagnosis of TC to death from any causes. Patients were censored at the time of the last visit until January 1, 2020. The study was approved by the Institutional Review Board of SNUH and SNUBH (IRB No. H-1703-035-836, B-1711–432-401).

### Next-generation sequencing

DNA was extracted from 10-μm thick paraffin sections containing a representative portion of each tumor block by proteinase-K digestion. Genomic DNA was isolated from FFPE tumor tissue blocks using the QIAamp DNA mini kit (Qiagen, Manchester, UK), and the qualified DNA samples were captured and sequenced with SureSelect (Agilent, Inc., USA) following the manufacturer’s instructions. The panel of 83 targeted cancer genes were focused on well-known oncogenes reported in the Catalog of Somatic Mutations in Cancer (COSMIC) database rather than relatively unknown genes whose functional effects are currently in question. The panel included the coding exons of 72 genes to detect single nucleotide variants (SNVs), insertion/deletions (indels), and copy number variations (CNVs), as well as some introns for 5 genes to detect gene fusions. After being aligned to the human genome 19 reference and filtered for germline polymorphisms and false positives, SNVs with a variant allele frequency ≥ 5% and indels with a variant allele frequency ≥ 10% were selected as the final results. SNVs and indels were detected with the ensemble method integrating three open-source callers: UnifiedGenotyler [9], LoFreq [10], SNVer [11], and SamsungSDS’s in-house callers.

CNVs were analyzed using the depth of coverage for each target region between the tumor and preprocessed normal data. For translocation detection, a paired-end mapping analysis and a split-alignment analysis were applied. All discordant read-pairs with an abnormal insert-size or orientation were screened and soft-clipping information of the split-reads was investigated as the evidence of the genomic rearrangements. The cut-off values used were CNs ≥7 for amplification, CN 0 for homo-deletion, and split-read support count ≥3 for translocation. SNVs, indels, CNVs, and translocations were discovered with in-house callers developed by SamsungSDS.

### Use of public database as a reference

We used gene expression data identified from panel sequencing to select possible functional genetic changes. Because TC is a rare disease without easy-to-obtain control samples, we used a public database as a reference. We searched the genetic changes identified in our samples in COSMIC (https://cancer.sanger.ac.uk/cosmic) and cBioPortal (http://www.cbioportal.org/public-portal/). All genetic changes identified in our samples were searched in Google Scholar to determine their significance. Genetic alterations were considered significant when referred to more than once in the main text or table of the article, showing the association with cancer development as a somatic mutation. Survival data was obtained from the Ministry of the Interior and Safety in South Korea for research purpose.

## Results

Four patients (50% female; mean age, 51.5 years) diagnosed with TC were identified with available FFPE tumor tissue blocks to extract DNA for analysis. The tumors were located in the head and neck of 3 patients and the shoulder of one patient. The mean overall survival (OS) was 88 months (range, 27–128 months). The mean coverage of all the samples was 286 (range, 32–679) (Supplementary Methods). *TP53* mutations (p.Arg213*, p.Arg249Trp and p.Arg248Gln) were found in three patients. Fusions previously identified in melanoma were detected in two patients (*TACC3-FGFR3* and *ROS1-GOPC* fusion). Other identified mutations included an NF1-truncating mutation (Arg1362*), an *NRAS* mutation (p.Gln61Lys), *TOP1* amplification, and the *PTEN* deletion. Among four patients, one experienced relapse; the tumor harbored a SNV in *NF1* (Arg1362*) and *ROS1-GOPC* fusion. The recurred tumor was not available for analysis because the quality of the FFPE tissue block was low lacking DNA. *TP53* mutations were identified in three patients. Patients with *TP53* mutations had an aggressive clinical course: two patients died (OS 93 and 27 months) and another experienced recurrent relapse. Overall, genetic changes found in TC resembled that of other skin cancers, suggesting similar pathogenesis (Tables 1, 2, 3, 4).

**Table 1:** Patient characteristics

Case	Sex	Age	Location	Mean target coverage	Tumor depth	Tumor size (cm)	Underlying disease	OS (months)	Survival status	DFS (months)	Relapse
1	F	36–40	Postauricular area	278.9	Dermis	1.4 × 1.2 × 0.6		128	Alive	128	No
2	M	41–45	Shoulder	32.2	Dermis	9 × 5 × 1.7		93	Dead	39	No
3	F	46–50	Scalp	151.7	Subcutis	1 × 0.8 × 0.3	Epidermodysplasia verruciformis Chronic hepatitis B CIN	124	Alive	33	Yes
4	M	71–75	Temporal area	679.9	Bone	5.2 × 5.0 × 3.3	HTN Osteomyelitis of thumb Cataract BPH	27	Dead	15	Yes

*CIN* Cervical intraepithelial neoplasia, *HTN* Hypertension, *BPH* Benign prostate hyperplasia, *OS* Overall survival, *DFS* Disease-free survival

**Table 2:** Genes showing non-synonymous somatic SNVs

Case	Chr	Position	Ref	Alt	Transcript	Gene	Effect	AA Change	VAF
1	17	29,576,111	C	T	NM_001042492.2	NF1	STOP_GAINED	p.Arg1362*	2.5
2	1	115,256,530	G	T	NM_002524.4	NRAS	NONSYNONYMOUS CODING	p.Gln61Lys	36.8
2	17	7,578,212	G	A	NM_001126112.2	TP53	STOP_GAINED	p.Arg213*	57.1
3	17	7,577,536	T	A	NM_001126112.2	TP53	NONSYNONYMOUS CODING	p.Arg249Trp	80.5
4	17	7,577,538	C	T	NM_001126112.2	TP53	NONSYNONYMOUS CODING	p.Arg248Gln	13.9

**Table 3:** Genes with copy number alterations which are well known to be associated with cancer development and/or progression

Case	Chr	Start	End	Gene	Genetic alteration	
2	Chr10	89,624,156	89,725,266	PTEN	Del	TC was reported in patients with Cowden syndrome, which is caused by germline PTEN deletion [6]. PTEN mutation is found in glioblastoma, endometrial carcinoma, lymphoma, thyroid, breast, prostate carcinoma, melanoma [12].
3	Chr20	39,657,593	39,752,015	TOP1	Amp	TOP1 amplification was reported in advanced and poor prognostic tumors in melanoma [13].

*Chr* Chromosome, *Del* Deletion, *Amp* Amplification, *TC* Trichilemmal carcinoma

**Table 4:** Fusion genes with possible functional change (Fusion between gene 1 and gene 2 were observed)

Case	Mean distance	Gene 1				Gene 2				
		Gene	Cytoband	Breakpoint	Transcript	Gene	Cytoband	Breakpoint	Transcript	
1	230,509	ROS1	6q21-q22	Chr6: 117639431	NM_002944	GOPC	6q21	Chr6: 117913860	NM_020399	Glioblastoma, Cholangiocarcinoma, ovarian cancer, NSCLC [14]
2	68,411	TACC3	4p16.3	Chr4: 1736923	NM_006342	FGFR3	4p16.3	Chr4: 1806178	NM_000142	Glioblastoma, bladder urothelial tumors, nasopharyngeal carcinoma, head and neck cancer, cervical cancer [15]

*NSCLC* Non-small cell lung cancer

### Case 1 (woman, 36–40): Postauricular TC with NF1 mutation and ROS1 fusion

A female patient presented with a recurrent mass at the postauricular area after laser therapy a year ago. Histology was consistent with trichilemmal carcinoma and invasion to reticular dermis. Perineural invasion or angiolymphatic invasion was not observed, and resection margins were free from tumor cells. Her TC sample from the recurrent lesion was positive for the *NF1* gene mutation (p.Arg1362*) and the *ROS1-GOPC* fusion gene. However, the FFPE tissue block from the primary tumor was not available for DNA extraction. She was free from recurrence for 6 years but underwent right upper lobe lobectomy for adenocarcinoma of lung (Stage IA1) after 5 years.

### Case 2 (male, 41–45): shoulder TC with FGFR3 fusion, PTEN deletion, and NRAS and TP53 mutations

A male patient visited the clinic with a growing shoulder mass that he first noticed 2 years ago. The mass (9 × 5 × 1.7 cm) was erythematous and verrucous, accompanied by spontaneous bleeding. The clinical impression was keratoacanthoma or verrucous carcinoma. Histological assessment of the mass indicated dermal invasion. Genetic testing identified the *TACC3-FGFR3* fusion gene, *PTEN* deletion, and the *NRAS* (p.Gln61Lys) and *TP53* (p.Arg213*) mutations. Disease-free survival was 39 months, and the patient expired with an overall survival of 93 months.

### Case 3 (female, 46–50): scalp TC with TP53 mutation and TOP1 amplification

A female patient presented with an exudative nodular lesion of the scalp. She had underlying epidermodysplasia verruciformis (EV), with multiple verruca plana on her whole body including the scalp. In addition, she had a history of chronic hepatitis B and cervical intraepithelial neoplasia. For EV, she had been treated with topical imiquimod, interferon-2α and acitretin or zinc. The exophytic exudative scalp tumor was diagnosed as trichilemmal carcinoma and showed clear resection margins. She experienced recurrence in multiple regions (scalp and bilateral postauricular area) 33 months after the initial operation. Another recurrent tumor was observed in the scalp and lower leg 7 years after the second operation. She did not undergo adjuvant therapy. The sequencing was performed from the initial specimen, and *TP53* mutation (p.Arg249Trp) and *TOP1* gene amplification was identified.

### Case 4 (male, 71–75): orbital TC with TP53 mutation

A male patient showed a mass in the right orbital region. He had underlying hypertension, benign prostate hypertrophy, osteomyelitis of the right thumb, and a history of cataracts surgery in the right eye. The mass was identified in the right frontal and superolateral periorbital area. Grossly, the tumor presented with slight protrusion of the temporal region without ulceration. Magnetic resonance imaging revealed that the lesion had invaded the temporalis muscle with extension into the lateral extraconal orbital cavity, abutting the lateral rectus muscle and involving the right frontal skull and lateral orbital rim. Histological assessment showed trichilemmal keratinization, consistent with trichilemmal carcinoma. Genetic testing of the tumor tissue identified a *TP53* mutation (p.Arg248Gln). The patient expired with distant metastasis with overall survival of 27 months.

## Discussion

TC is a rare malignant adnexal neoplasm originating from the outer root sheath, wherein cells demonstrate continuity with epidermis or follicular epithelium [5]. Although the pathogenesis of TC has not been clearly identified, it appears to be related to actinic damage, long-term low-dose irradiation, or malignant transformation of trichilemmoma. Histological assessment of TC reveals varying degrees of local actinic damage. Oyama et al. [7] reported trichilemmal carcinoma from long-standing seborrheic keratosis. In addition, other reports have described TC cases in patients with xeroderma pigmentosum or Cowden syndrome [6], as well as in solid-organ transplant recipients. Some of the genetic alterations identified from our samples are in line with previous reports, enabling us to suggest possible pathogenetic mechanisms of TC.

Takata et al. [8] reported that the total loss of the tumor suppressor gene *TP53* may result in malignant transformation in TC. The complete loss of wild-type p53 due to the allelic loss of the short arm of chromosome 17p was suggested as a critical event. *TP53* mutations were observed in three samples from our study, supporting the role of *TP53* mutations in TC occurrence. *PTEN* deletion was identified in Case 2, but the patient did not have Cowden syndrome. Cowden syndrome, also called PTEN hamartoma syndrome, is caused by germline *PTEN* mutation. It develops multiple trichilemmoma, which is known to be a premalignant lesion of TC. O’Hare et al. [6] reported a patient with Cowden disease who developed TC. Acquired *PTEN* deletion in Case 2 may have played an important role in the development of TC, possibly from trichilemmoma. *TOP1* amplification was identified in one patient with underlying EV. EV is characterized by persistent human papillomavirus (HPV) infections, especially the ß-HPV subtype including HPV-5 and HPV-8. In addition, EV is prone to developing skin cancers [16], most of which contain HPV [17, 18]. The E6 protein from HPV-5 and HPV-8 decreases the level of ATM serine/threonine protein kinase through p300/ATR signaling, increasing carcinogenic potential after UV exposure [19, 20]. However, *TOP1* inhibitor is reported to be effective for ATM mutations. We hypothesize that in this patient, decreased ATM caused by HPV resulted in the carcinogenesis of TC. In such circumstances, *TOP1* inhibitor may be used as therapeutic agents. In addition, the association with HPV has been reported in other genetic alterations. *FGFR3-TACC3* fusions, found in various solid tumors, were identified in tissues of head and neck squamous cell carcinoma that were also positive for HPV [14]. Moreover, *PTEN* deletion, identified in Case 2, frequently accompanied HPV-induced squamous cell carcinoma [21]. These findings imply that HPV may play a leading role in TC carcinogenesis.

Our TC cases showed common genetic variants with skin cancers. *TP53*, *NF1*, and *NRAS* are frequently mutated genes identified in malignant melanoma [22]. *TP53* mutations in melanoma are correlated with sun exposure [23, 24]. In addition, *TOP1* amplification was reported to be associated with more advanced and poor prognostic tumors in melanoma [12]. Furthermore, *TP53* and *NRAS* mutations have been identified in squamous cell carcinoma. Various skin cancers are reported to develop from seborrheic keratosis; one report reviewed a patient who developed TC in long-standing seborrheic keratosis [7]. Given the shared risk factor, chronic sun exposure, TC may have similar molecular pathogenesis with skin cancers. Although controversial, some reports support the origin of basal cell carcinoma as outer root sheath of hair follicle, similar to TC [25, 26]. This also suggests that carcinogenesis can be induced in outer root sheath cells by UV damage. Despite different cells of origin resulting in different types of cancer, a common carcinogen may cause the same genetic mutation for carcinogenesis.

*ROS1-GOPC* fusions are well known to be found in glioblastoma, cholangiocarcinoma, ovarian cancer, and non–small cell lung cancer. *FGFR3-TACC3* fusions have been reported in glioblastoma, bladder urothelial tumors, nasopharyngeal carcinoma, head and neck cancer, and cervical cancer. Moreover, *FGFR3-TACC3* [27] and *ROS1-GOPC* fusions [28] were reported in melanoma. For both cases, targeted agents showing therapeutic responses to the respective fusions were introduced. A recent report described a patient with metastatic melanoma harboring *GOPC-ROS1* gene fusion who showed clinical response to entrectinib [28]. Therapeutic responses can also be expected in TC cases after the identification of such fusion genes.

Our study nevertheless has several limitations. First, due to its retrospective design, the study was susceptible to selection bias. Of note, we could not obtain detailed follow-up data of case 2: although survival outcome was available from the Ministry of the Interior and Safety in South Korea, cause of death could not be identified. Second, we extracted DNA from FFPE tissue blocks, and fixation and storage process may have caused artifacts and low quality. Third, because we used a gene panel, genes not included in our panel could not be tested, possibly leading to significant implications. Fourth, we did not assess the functional consequences of the identified genetic mutations. Fifth, due to the limited sample size, statistical analysis was not possible. However, due to the rareness of the disease, our findings bear significance. Further studies are warranted to investigate certain molecular pathogenesis of TC and related therapeutic and prognostic implications.

## Conclusions

We reported the genomic variations found in TC, which may give insight into the molecular pathogenesis. Overall, genetic changes found in TC resembled that of other skin cancers, suggesting similar pathogenesis. TP53 mutations was were identified in patients who had an aggressive clinical course. Genetic alterations identified may further suggest the potential treatment options of TC.

## Data Availability

The datasets generated and/or analyzed during the current study are available in the SRA repository, [https://www.ncbi.nlm.nih.gov/sra/PRJNA613537].

## Abbreviations

TC: Trichilemmal carcinoma
FFPE: Formalin-fixed paraffin-embedded
COSMIC: Catalog of Somatic Mutations in Cancer
SNV: Single nucleotide variants
CNV: Copy number variations
OS: Overall survival
EV: Epidermodysplasia verruciformis
HPV: Human papillomavirus
